# Slippery Knowledge: Ignorance, Ecologies, and Environment in Endometriosis Framing

**DOI:** 10.1111/maq.70002

**Published:** 2025-05-20

**Authors:** Andrea Ford

**Affiliations:** ^1^ Centre for Biomedicine, Self, and Society University of Edinburgh Edinburgh UK

## Abstract

Despite a growing body of literature linking environmental toxins and endometriosis, environmental issues make only occasional appearances in public, patient, and specialist conversations about endometriosis. These conversations may hover at the edges of public discourse, but do not gain traction. Based on ethnographic work in the United Kingdom, this article develops the concept of “slippery” knowledge as that which evades action. Ignorance of environmental or ecological etiologies is less a dearth of information than a dearth of possibilities for action. This article elaborates two ways of conceiving of environmental or ecological disease: the exposure model predicated on harmful external factors “getting in” to damage individuals or communities and the embodied ecologies model, which posits inevitable and ongoing mutual imbrication among living and non‐living entities. Knowledge regarding endometriosis is “slippery” in both models. Whether knowledge seems actionable or not is inextricable from deep‐seated power dynamics related to colonialism, gender, and race, which perpetuate ways of knowing (and acting) on endometriosis that are troubling and troublingly durable.

We sat on a log in the Mortonhall arboretum in Edinburgh, occasionally waving away the midges that hovered to bite our ankles. My old dog accompanied us. The grounds of a manor‐turned‐public‐park was an unconventional site for an interview, but I had become friends with Diana over the course of shadowing the endometriosis support group she led. The COVID‐19 lockdown had been briefly lifted by the Scottish government and we were eager to be outside. Diana shared how she retrospectively linked experiences like migraines and morning sickness with her menstrual cycle after receiving a diagnosis of endometriosis at age 33, roughly 20 years after she started experiencing difficulties during her cycles. Her story was utterly typical in that she was told period pain was normal, was prescribed birth control pills, and felt dismissed by doctors who “found nothing wrong.” She exclaimed, “I can't believe that it actually took for me to faint in an airport because of the pain to actually understand that okay, this is not normal.” I mentioned the midge bites at one point and she quipped, “Oh, I don't feel them. You see this is one of the pluses of endo… you're like, normal pain is just, irrelevant.”

Her story elaborated on the harrowing and, by now, well‐reported patient narrative in how she coped with the common chronic disorder by connecting it to factors in her personal life. After she mentioned the migraines, I asked her to tell me more about how she manages her cycle and how it affects her life. She was clear that the cycle wasn't the problem; the pain was. The cycle she could “build a relationship with,” and she explained her appreciation for her cyclical energy levels, sensitivities, and kinds of perception, crediting endometriosis with prompting her to become aware of this rhythm. However, making some kind of master plan of cyclical symptom management was elusive: “we're just such complex creatures, you know? I mean, everything makes a difference—what you eat, what you drink, how much you sleep, how much stress you take in… I'd need some sort of data warehouse to map all those things out. So, it's hard to map everything. But I'm trying to work it out.” She was a computer programmer and no stranger to complex intellectual problems.

As our interview progressed, she elaborated on how such a “map” would need to include far more than her personal habits. I asked about hormones, and she shared that she understood the disease to be exacerbated by estrogen (scientific consensus agrees; Taylor et al., 2021). But she immediately leapt to a less‐common variant of the hormonal rationale. “In today's world we are surrounded by man‐made estrogens,” an idea given to her by her surgeon. “He was the one who put all these thoughts in my head,” she shared, telling me about his research inducing endometriosis in lab rats by exposing mother rats to estrogens prior to conception. But while she was initially enthusiastic about the surgeon's ideas, she began to have doubts about the theory's explanatory power. “Why don't other people get [endometriosis]? I don't think it's fully that.” She talked about genes and prenatal trauma, using her own story to elaborate possible transpersonal explanations: “I'm talking not just about my life, I'm talking about generations and patterns that… could play a role. I can't prove that. Who can prove it?” Such an explanation would be “not just about you, it's about you and the environment in which you operate. And that environment has its own history.” She discussed the devaluation of women and the Earth, the denigration of the feminine everywhere from sexuality to industrial farming of eggs and milk. When I offered the summary, “It's everything,” she nodded and added, “And everything is broken right now, I think. And we need to balance it somehow. And I don't know how that would be possible.” These expansive environmental themes stood out to me for how unusual they were throughout my fieldwork.

## INTRODUCTION

Where does the environment begin and end? As we can see in Diana's account, this question has temporal, spatial, and social dimensions. Philosophically speaking, the very concept of “an environment” is fraught, as it presumes a coherent subject set apart from its background, sidestepping the many ways human individuals are not just surrounded by, but *composed of*, living and non‐living entities. This is what Formosinho et al. (2022) describe as “environmentality,” wherein one is an environment for something else, and which I have called “embodied ecologies” (Ford, 2019). Such complexity is not usually centered in considerations of environmental health and environmental etiologies, which are themselves marginalized frames for understanding (human) disease (e.g., Allen, 2008; Brown et al., 2011; Kroll‐Smith et al., 2000; Nash, 2006; Workneh et al., 2024). Despite a growing body of literature showing that there are strong connections between environmental toxins and endometriosis etiology (Dutta et al., 2023; Rumph et al., 2020; Sirohi et al., 2021; Wen et al., 2019), endometriosis is not usually considered an environmental disease. Environmental links and resonances regarding endometriosis make occasional appearances in public and patient conversations as well as specialist medical conversations but are far from central in current debates and campaigns. Instead, these ideas hover at the edges, but do not gain traction. This article addresses this omission, or “slipperiness.” Absent knowledge can be the object of social science, as a growing literature of ignorance studies demonstrates (Gross & McGoey, 2015; Scott, 2018; Tuana & Sullivan, 2006)—a legacy stretching back to feminist standpoint theory in the late 20th century and Marx and Freud's 19th‐century accounts of systematic interested ignorance (Harding, 2006). What is not found, not known, not talked about, and not looked for is as politically determined and, often, as important as what is.

Endometriosis is a common, chronic, systemic inflammatory condition characterized by debilitating pain, fatigue, and issues with infertility, and troubled by 7–12 year‐long delays in diagnosis (and growing: Endometriosis UK, 2024). Other issues include difficulties with communication between clinicians and patients, lack of effective treatments, and shame and stigma both within and outside the clinic. Despite affecting around 10% of menstruating people, the same proportion as diabetes or Crohn's disease, and at least 176 million individuals worldwide, endometriosis is under‐researched and its etiology poorly understood (for authoritative medical reviews, see Horne & Missmer, 2022; Taylor et al., 2021). A recent call decried the slow pace of research progress and emphasized the need for “a sweeping and revolutionary breakthrough,” stating that “if there is no change in the way we conceptualize the disease, no matter how much more data we accumulate and/or how we acquire these data, it will add little to our understanding” (EIG, 2024, 372).

Endometriosis is often described as “enigmatic,” a form of ignorance that Nicky Hudson shows is *produced* and not an inevitable feature of the condition (2022). She illustrates how endometriosis is a “missed disease” and an example of “undone science” in her eponymous piece. In her words, “ambiguity around endometriosis is part of a wider constellation of discursive, material and political factors which enroll certain forms of knowledge whilst silencing, ignoring or marginalizing other forms of knowledge” (Hudson, 2022, 21). Hudson argues that ignorance has become key to what endometriosis is understood to be: “The claiming of this (non)knowledge about the disease by scientists demonstrates how a description of endometriosis as enigmatic is performed as a characteristic of the disease itself, rather than a failure of science to fully comprehend its nature” (Ibid., 24; see also Seear, 2014). I see the elusiveness of “environment” in efforts to understand, treat, and discuss endometriosis as a subset of this broader phenomenon, sometimes aligned with and sometimes at odds with other currents within this ignorance.

I was interested in how environmental themes were showing up for clinicians and patients when I designed and conducted my ethnographic study of endometriosis in and around Edinburgh. It turned out that environmental themes weren't showing up, really. Most people I spoke with only mentioned environmental factors in response to direct questioning, and many had nothing to say on the topic. Initially, I found this discouraging—a “null result”—though absences can speak loudly if attended to with care. Diana was a conspicuous exception to this trend, and I find her account useful because of the way it slides through increasingly expansive versions of what “environment” means—a scalar ambiguity that contributes to environment's marginalization.

In this article, I argue that environmental concerns are elided because differently situated people feel powerless to act on environmental etiologies. This happens in nested ways, in which at least two conceptions of environment are at work. The first conception of environment is the “exposures” model, which posits an individual on a (toxic) background that might “get in” and should therefore be defended against. This leads to logics of consumer politics and health behaviors, which are disempowering for both patients and clinicians because they are hard to enact given different patients’ affordances in the social world and clinicians’ lack of preparation for talking with patients about these issues. These sociological dynamics are exacerbated by commercial interests invested in producing both toxins and ignorance about their effects (see Lancet, 2023).

The second conception of environment is the “embodied ecologies” model, in which body boundaries are understood to be porous such that bodies are inextricable from what surrounds and composes them, and effects take place across lengthy, intergenerational timespans. Embodied ecologies add to existing concerns regarding consumer politics and health behaviors as insufficient and disempowering by suggesting that preventing exposure for given individuals or populations is illusory, and seeking protection in this way is perhaps pointless. Within this model, bounded individuals don't exist (unthinkable within a biomedical context). Thus, environmental illness troubles the epistemologies and ontologies of scientifically knowable bodies, and enacting environmental concerns within this register is not just practically difficult but conceptually difficult.

In what follows, I first lay out the links between toxins and endometriosis, explain my research methods, and elaborate the conceptual difference between an environment and an ecosystem. Then, I discuss the difficulties of acting on environmental/ecological knowledge, first within the exposure model and then the embodied ecologies model, including an analysis of the environmental themes that came up in my interviews. Finally, I discuss “slipperiness” as a form of knowing/ignorance in which ways of understanding that are difficult to act upon are allowed to slip away, while individualizing, misogynistic, and colonial ways of understanding are troublingly durable. This is not an attribution of intention or values to particular actors but an attempt to articulate how taken‐for‐granted knowledge practices are reliant upon deep‐seated power dynamics that elide other ways of apprehending the world.

## WHAT HAS ENVIRONMENT GOT TO DO WITH IT?

It has been over 20 years since Stella Čapek published a chapter titled “Reframing endometriosis: from ‘career woman's disease’ to environment/body connections” in a volume on environment and illness (Čapek, 2000). This was one of the earliest pieces in an exponentially growing set of critical, feminist, and patient‐oriented publications on endometriosis (e.g., Bullo & Weckesser, 2021; de C. Williams & McGrigor, 2024; Griffith, 2017, 2020; Jones, 2016; Seear, 2014; Young et al., 2019). Čapek's piece documents the efforts of an American patient advocacy organization, the Endometriosis Association (EA), to complicate, politicize, and raise awareness regarding endometriosis within conventional biomedicine throughout the 1990s, a mission that has likewise steadily gained international traction. In Čapek's account, the EA combatted dismissive gendered nonsense like the idea that “career women” are susceptible to endometriosis because they have shunned their reproductive purpose; instead, the EA emphasized strikingly conclusive research about transgenerational exposure to dioxins (chemical toxins) causing endometriosis in monkeys. Čapek, and apparently the EA organizers of that time, thought that this had fundamentally shifted how endometriosis should be framed. Yet today, endometriosis is hardly considered an environmental disease. The “successful redefinition” declared in the piece (2000, 346) is, at best, premature.

Yet, recent systematic reviews continue to suggest that chemical toxins of various kinds are implicated in endometriosis etiology (Dutta et al. 2023; Rumph et al., 2020; Sirohi et al., 2021; Wen et al., 2019). These studies focus on EDCs (endocrine disrupting chemicals), synthetic chemicals that mimic or block hormone function and are ubiquitous in manufactured goods and post‐industrial landscapes. The most recent of these reviews states that “The available information strongly indicates that environmental exposure to EDCs such as PCBs, dioxins, BPA, and phthalate individually or collectively contribute to the pathophysiology of endometriosis” and that “these four EDCs activate multiple intracellular signaling pathways associated with proinflammation, estrogen, progesterone, prostaglandins, cell survival, apoptosis, migration, invasion, and growth of endometriosis,” calling for further research into the molecular mechanisms (Dutta et al., 2023, 56).

UC San Francisco's Program for Reproductive Health and the Environment is an academic medical center investigating this nexus. They state that the risk of developing endometriosis is about 50% genetic and 50% environmental (PRHE, 2023). Similar to the dioxin studies Čapek hailed as transformative, recent research shows that children born to parents with a history of exposure to manmade toxic agents, notably dioxins, are at heightened risk of developing endometriosis (Rumph et al., 2020). Researchers at Vanderbilt University's Medical Center claim that environmental triggers of inflammation and endometriosis induce epigenetic changes that persist across *five* generations (VUMC, 2023; see also VUMC, 2020). They see this as key to preventing endometriosis instead of reacting to it, referring to toxicant exposure as a “root cause.” Furthermore, they link the disproportionate exposure of people of color to endocrine‐disrupting chemicals, persistent organic pollutants, and heavy metals to racial disparities in women's health conditions (Rumph et al., 2022).

When I moved to the United Kingdom for an academic position in 2019, I was intrigued by these connections (or earlier intimations of them, as much of the literature referenced here is more recent). I conducted fieldwork based in and around Edinburgh consisting of in‐depth interviews with clinicians and clinical staff (6) and people with endometriosis or suspected endometriosis (13) who ranged in age from early twenties to septuagenarians during 2020–2021, as well as attending local support groups. Ethical approval for the study was sought and obtained from the East of Scotland Research Ethics Service, interviewees provided written informed consent, and participants are referred to by pseudonyms in this article. I also attended the 2023 World Congress on Endometriosis, participated in government and policy engagement, followed online forums throughout 2019–2024 and was involved in organizing a high‐profile “Reframing Endometriosis” British Academy conference. I consider medical and social science literature to be a “field site,” too. Although I began the study curious about how environmental thinking was circulating among patients and clinicians, I found that endometriosis was not usually approached from environmental, ecological, or toxicity‐linked angles across these field sites. In this piece, I am taking a large step back to look at the context that makes environmental toxicity so elusive.

In many ways, elision of environmental causes is unsurprising; the predominant emphasis in biomedical and even public health disciplines is ever‐more resolutely on individual bodies and behaviors, a depoliticized and decontextualized version of health and disease (for canonical examples, see Crawford on healthism [1980] and Rose on responsibilization [2007]). Environmental health was foundational to the origins of public health in the 19th century, expressed in concerns with “miasma” (bad air), water supply, sanitation, and industrial pollution; yet, concerns of this nature have become less central as much public health work focuses on behavior change across populations (see Scally, 2014). After World War II, the boom in manufactured chemicals was largely heralded as emblematic of scientific progress and healthier modern living. Skepticism about their beneficence is still shrouded in marginalizing adjectives like “natural” and “alternative.” Unlike infectious disease and hygiene, which are staples of epidemiology, *chemical* etiologies of disease are notoriously hard to study and establish beyond correlation.

Much linkage between environmental toxins and human health comes from outside of medicine proper. Decades of work in medical anthropology and allied disciplines have argued for the importance, indeed centrality, of socioenvironmental context to health and disease. Environmental justice activism draws attention to environmental toxicities and their differential burdens (but universal implications) (e.g., Hoover, 2018; Liboiron et al., 2018; Nixon, 2011), including reproductive environmental justice, a framework originating from Indigenous scholars (e.g., Dow & Chaparro‐Buitrago, 2023; Lappé et al., 2019; Liddel & Kington, 2021). One recent “toxic autobiography” in this vein connects endometriosis, pollution, climate change, and disability (Iengo, 2022). Citizen science initiatives to measure exposures, hypothesize about cause, and advocate for change are proliferating (e.g., Brown et al., 2011; Corburn, 2005; Hess, 2016; Shapiro, 2015), including a more expansive “canary science” (Grandia, 2021), referring to the adage about the “canary in the coalmine” who is the first to suffer toxic effects. Approaches to health and healing labeled “alternative” often engage with issues of toxicity and an expansive view of disease as “everything out of balance,” as Diana articulated, including popular movements to balance hormones through lifestyle—however, extra‐medical approaches to healing can easily slip into individual responsibility and self‐blame if inadequately politicized, as has happened with endometriosis “self‐management” (Seear, 2014). The relative marginalization across all these forms of extra‐medical knowing speaks to their “slipperiness.”

Pervasive uncertainty about what endometriosis is and how it works makes it interesting and relevant that environmental etiologies are sidelined. If there were solid scientific consensus about disease mechanisms and treatments, pointing out the absence of a particular line of thinking would require more justification. It is useful to spend some time explaining endometriosis here, as despite patient and physician advocacy success over the past two decades, shame and stigma keep it obscure (and exacerbate its harms in a “syndemic” [Griffith, 2017]). The condition occurs when tissue similar to the uterine lining (the endometrium) develops outside the uterus; this hormonally responsive tissue “bleeds” during menstruation, forms lesions of scar tissue that can fuse organs and internal tissues together, and often causes chronic pain. A clinician I interviewed described it as “mini periods outside the womb.” It affects people differently, and the presence and severity of lesions do not correlate with the presence and severity of pain, which is multifaceted and difficult to communicate (Bullo & Weckesser, 2021). If lesions form near the ovaries or fallopian tubes, they can impact fertility; if they form near the intestines, symptoms can resemble irritable bowel syndrome; if they form around the vagina, pain during sex can result. Lesions usually form throughout the pelvis but have been found in the lungs, brain, and knees. Most people who experience endometriosis are women, but trans men and non‐binary people do as well, alongside a tiny number of cis men (Al‐Obaidy & Idrees, 2019).

The “gold standard” of treatment involves surgically removing the lesions, although this doesn't always relieve symptoms (and rarely does so permanently) and may even make them worse. Symptoms can persist after a total hysterectomy, in which the uterus and ovaries are removed. The other accepted treatment is “medical management” (pharmaceutical symptom suppression) using synthetic hormones and/or painkillers. Regularly taking analgesic drugs raises complex issues of desensitization and dependency, alongside the fact that endometriosis pain can become “neuropathic,” or learned and perpetuated by the nervous system itself. Hormonal medications, which either stop ovulation (birth control pills) or prematurely induce menopause, aren't an option if one is trying to conceive and can mask symptoms of endometriosis until this point. Some clinicians I spoke with noted that hormonal medication's success at suppressing endometriosis symptoms contributes to delayed diagnoses, in effect generating outrage over an effective treatment (Interestingly, there is no reason not to consider hormonal drugs as “endocrine disrupting chemicals” except that they are intentionally taken into one's body and have supposedly predictable effects).

Technically, the disease *is* the lesions of out‐of‐place tissue, and diagnosis involves finding them surgically, which contributes to the delay. There is widespread dissatisfaction with this lesion‐centric definition of the disease, with some physicians calling for endometriosis to be considered a symptom‐centric “syndrome” instead of a disease (Hickey et al., 2020). Patients have been hesitant about the lack of legitimacy associated with a “syndrome”—indeed, a diagnosis is often embraced for offering legitimacy and clarity (e.g., Ballard et al., 2006), even though it doesn't necessarily lead to any relevant treatment. Griffith (2020) calls this the “a‐diagnostic category” in which patients have “been given an explanation for their symptoms but such explanations remain non‐medicalized such that subsequent treatment is not given” (2020, 32). There is a growing medical consensus that endometriosis should be considered a whole‐body, systemic condition, instead of a (solely) gynecological one. Taylor et al. describe how endometriosis affects metabolism, leads to systemic inflammation, and alters gene expression in the brain that causes pain sensitization and mood disorders. They write, “The full effect of the disease is not fully recognized and goes far beyond the pelvis” (2021, 839).

Considering this uncertain state of knowledge and treatment, there are abundant issues about which people might be concerned. My interviews with both patients and providers are full of passion and pain, injustice and outrage, good intentions and care. And yet, much of what was said by patients echoed what has been documented and analyzed before: stigma, dismissal, runaround, gaslighting, and isolation, alongside the importance of supportive communities and resigned attempts at self‐management. There is a “stuckness” in the experience of having endometriosis. “That could have been yesterday!” exclaimed an audience member at the “Reframing Endometriosis” conference in 2023 after Elaine Denny, one of the first social scientists to study endometriosis, showed quotes from her interviews at the turn of the century. There were murmurs of agreement and exasperation. Little has changed. This paper attempts to step outside the entrenched narratives to gather some perspective on possible alternatives.

## THE EXPOSURE MODEL

The systematic review quoted above advocates for further research to “help regulate the exposure to these EDCs in reproductive age women” (Dutta et al., 2023, 56). The exposure model presumes that individuals are relatively clean slates who are contaminated by their environments through “exposures” to what is harmful. This model of environment tends to be the default in the United Kingdom and other liberal democracies, predicated on individuals as the basic units of society, as opposed to a more holistic understanding. I have elsewhere defined “environment” and “ecosystem” in relation to health (SAPEA, 2024); while the latter is a holistic system comprised of living and non‐living entities that are both interconnected and interdependent, the term environment perpetuates the idea that a person (or sometimes an animal or a species) is the “figure” on an environmental “background” or surrounded by an environmental “context,”—meaning that the environment is a *place* instead of a collective of entities and processes in which humans are implicated. Importantly, humans are *part of* ecosystems. Ecosystems are plural and localized but also interconnected with and interdependent on each other such that the planet can also be described as an ecosystem. The term “nature” has similar problems to “environment”: it usually implies places and processes that are separated out from human activity, which is an untenable premise. The exposure model problematizes the environment as full of dangers that might invade or compromise the individual. Toxicological and epidemiological research describes this as the “exposome,” an environmental complement to the “genome” (Wild, 2005).

This framing resonates with traditional ways of thinking about immunity as protection against what harms us, setting up a clear demarcation between what is “self” or internal versus “other” and external (a model of immunity being updated and challenged in various ways across medical and social sciences, shifting towards frames not of defense but negotiated co‐becoming; e.g., Ford & Swallow, 2024; Napier, 2012). As Eula Biss (2015) writes in her cultural history of immunity, “…fear of toxicity strikes me as an old anxiety with a new name. Where the word filth once suggested, with its moralist air, the evils of the flesh, the word toxic now condemns the chemical evils of our industrial world… both theories allow their subscribers to maintain a sense of control over their own health by pursuing personal purity” (2015, 75). Consumer “purity politics” is the dominant mode of response to toxicity. It can offer a sense of agency, yet is uncomfortably moralizing, structured by existing power relations and inequalities, and relatively impotent given the ways toxicity seeps throughout time and space (Ford, 2020). Buying non‐toxic products furthers neoliberal insistence on an individual's responsibility for their circumstances and market‐based solutions to social problems. It is exclusive because of the time and money it takes to pay for products, educate oneself, read labels, and change shopping routines (particularly in less affluent areas); it's also gendered, as women and mothers do most shopping labor for the household (MacKendrick, 2018).

In my interviews, a few people with endometriosis discussed purchasing organic food and cleaning products when I asked about environmental factors, even while recognizing the limits of this approach. Mary had heard of EDCs and mentioned xenoestrogens in a vague way:

[I know the term endocrine disruption] because I like to read up a lot about stuff, but again I've not found a lot of information… I'm a vegetarian and I very much try to be organic and stuff where possible… you can't obviously get rid of everything because it's all around you all the time. Even in teabags… you can't escape it. But wherever possible I've tried to cut out the crap, basically trying to have kinder [soaps] sometimes… there are all these xenoestrogens or something out there, that don't help with the brain.

Knowing what types of chemicals are present in which products is a daunting task. Specialist clinicians I spoke with were aware of endocrine disruption but found it tangential to their research and clinical practice. Indeed, what could a clinician advise a patient other than consumer recommendations they might not be able to implement, recommendations that could easily be too vague or too complex? A 2014 survey of US obstetricians found that, although the majority believed that asking about exposure to toxic chemicals would benefit their patients’ health, “they equated counseling with ‘opening Pandora's box’” because they lacked adequate understanding to answer patients’ questionsʼʼ (Sutton et al., 2016, 556). Chemical exposure exceeds the purview of traditional medical training, though some advocate changing curriculums (Kligler et al., 2021).

Regulatory advocacy is a less individualized way of mitigating toxic exposures. Activists urge scientific experts, medical doctors, and concerned citizens to lobby governments to limit chemical companies’ actions and clean up or contain contamination, facilitated by organizations such as Healthy Environment and Endocrine Disruptor Strategies (HEEDS), the Collaborative for Health and Environment (CHE), and Physicians for Social Responsibility (PSR). Because chemicals travel, regulatory and research initiatives should exceed individual countries (Le Moal et al., 2016); the International Federation of Gynecology and Obstetrics (FIGO) considers “Toxic Environmental Exposures” a key issue. Some clinicians consider regulatory and political action part of their remit and responsibility (e.g., Giudice et al., 2021; Sutton et al., 2016). Such advocacy can take a more targeted form, as in regulations on the materials that can be used to make products for babies, or the Dutta et al. review quoted above that aims to regulate EDC exposure “in reproductive age women.” These targeted forms of regulation fall within an exposure model, whereas more generalized and expansive regulatory aims tip towards an embodied ecologies model, such as pressing for a precautionary approach to chemical manufacture whereby safety must be proven prior to approval or the recent push for a Global Plastics Treaty that aims to radically curtail global plastics production. Here, it is worthwhile to note that targeted exposure‐based regulations tend to ascribe demographic risk that can go hand‐in‐hand with the surveillance and policing of women and with mothers being saddled with extra responsibility for their children's health (see MacKendrick, 2018).

Yet, powerful commercial interests promote the use of manufactured chemicals, complicating research and regulation. Čapek notes that the EA's shifting focus onto environment/body connections could create “new enemies,” such as chemical manufacturers, or at least, “a contradictory position when a pharmaceutical corporation… provides funding for its conferences and at the same time manufactures suspected carcinogenic pesticides” (2000, 357). The difficulty of taking action within an exposure model, then, is compounded by vested interests and the ignorance they perpetuate. But there is a deeper difficulty here as well, to which we will now turn: an ignorance located in how impossible it is to separate synthetic chemicals from our contemporary ways of living. Vincanne Adams (2022) traces the cultural history of the ubiquitous toxin glyphosate, notorious from one of its many lives as the herbicide Roundup; she argues that a simple politics of demonization and victimhood is inadequate to convey “a much larger problem faced by anyone living in the ruins of agrochemical industrialism” (2022, 5). Instead, she attempts to “shift our focus from abject suffering to wider questions about how we live with the chemicals that have become ubiquitous” (2022, 6), something Michelle Murphy has called “alterlife” (2017).

## THE EMBODIED ECOLOGIES MODEL

Rather than a model of defense and protection, of “mitigating exposure” in which an entity (including a nation, a population, or a species) is lifted out from among what it is connected to, some scholars argue that toxicity requires a totally different, non‐individualized, expansive (and infiltrating) approach to action (e.g., Liboiron et al., 2018; Shotwell, 2016). Against the dominance of an atomized view of the world, an ecological view presents deep intellectual challenges such that it is almost unthinkable (Lefkaditou & Stamou, 2006), much less actionable.

Yet, because endometriosis evades explanation via accepted frames of reference, the unthinkable persistently suggests itself. The “cut it out” thinking that has dominated body‐as‐machine biomedical approaches to endometriosis falters: surgically removing the lesions or the uterus has unreliable effects. Because the presence of lesions does not correlate with the sensation of pain, the lived experience of endometriosis challenges deeply ingrained ways of thinking about reliable evidence of disease—that is, evidence drawn from an external view of the material body instead of the accounts of those experiencing it (Foucault, 1976). Similarly, endometriosis challenges traditional medical specialties based around anatomical points of reference and discrete “internal systems” like the reproductive, immune, or nervous system; it evades conventional disease categories (Ford, 2024). This problem resonates with other poorly understood, chronic, systemic immune/inflammation conditions which one has to “fight to get” (Dumit, 2006), such as fibromyalgia, multiple chemical sensitivity (MCS), and chronic fatigue syndrome (ME/CFS), which predominantly affect women and are persistently feminized.

The exposure model is inadequate for researching the complexity of the exposome. Diffuse chemical causes and correlations are difficult to study because we are always exposed in mixtures, making it challenging to isolate variables and distinguish signals from noise (Gao, 2021). Chemicals are not individually “good” or “bad,” which is the premise of current regulatory models, but develop capacities for harm through their interactions and over time. There is no “pure state” unaffected by chemical toxicity to act as a control in experiments or studies, since the ways chemicals spread throughout space and remain latent across generations means that we are all already compromised (Murphy, 2017; Shotwell, 2016). In many instances, timing of exposure (such as during embryogenesis) is understood to matter more than the level of exposure, counter to the toxicological maxim “the dose makes the poison” (Soto & Sonnenschein, 2024). Controlled scenarios, such as recent “organ on a chip” research that creates, essentially, a lab version of a person's tissues, represent more specificity (or “personalization” in industry terms) in order to “identify at‐risk individuals earlier” to target them for nutritional or pharmacologic interventions that reduce the risk of developing endometriosis or other diseases (VUMC, 2020). This is not about defending against exposure per se but having a more complex picture of a given person's potentialities and vulnerabilities; it is still an individualized, anatomical, and reactive framing in which action is (potentially) enabled without comprehending or intervening in the whole. Thus, taking action within an embodied ecologies model requires a paradigm shift.

For example, while concerns about toxic environments tend to focus on chemicals and pollution, they might as well also include stress and (a dearth of) microbes. Stress and the microbiome are immunologically relevant. The “stress system” encompasses hormones and inflammation; some research suggests that formative trauma correlates strongly with endometriosis (Hawks et al., 2019), and that quotidian stress (including the stress of having endometriosis) exacerbates the disease, acting as both cause and consequence (Griffith, 2017; Reis et al., 2020). The gut‐brain axis of hormonal‐chemical‐microbial interactions shapes experience (including the neurological experience of pain), which has been the subject of biological as well as critical research (see Wilson, 2015). What exactly are the differences between an environmental disease, an experiential disease, and an inflammatory disease? Indeed, “inflammation” can be construed as a global condition introduced and perpetuated by colonial violence against embodied ecologies, inextricable from an abstracted, depoliticized view of medicine (Marya & Patel, 2021). Some regulatory initiatives seek to protect ecosystems in ways that transcend human‐specific concerns, such as the “rights of nature” movement and One Health policy. Ecological action invites cultivating mutual accountability and deep system change.

Registering possibilities for action is challenging within the blurry zone between the exposure and embodied ecologies models. The third and final interviewee with endometriosis who had thoughts about environmental correlations, Maya, briefly mentioned behaviors like diet and “health style” but was mostly concerned with stress:

I truly believe that, as with other diseases, there are environmental factors… this is why some people experience endo so differently, so it depends on your diet, on your health style, on different things, on your level of stress. And for me the trigger was when I was working in a very, very stressful environment and I was at my limit, and this is when I started to have these extreme cramps… it's not that we can get rid of stress totally, because it's the world we live in, and my career also is very demanding in this way. But I think we can find ways to try to manage this; so I quit my previous job and then we moved.

She continued to mention toxins in food and how she was trying to have a more organic diet because of pesticides. When I asked her to say a bit more about toxins in the environment, she quickly re‐emphasized stress:

…stress is the most terrible trigger, I think, because where I used to live before, in Brazil, it was not a safe place; so it's like your body is stressed all the time, like you were waiting for the danger… this creates a continual stress and continual inflammation in your body.

I asked her whether she considers things like stress or toxins as hormonal problems, and she replied that everything affects hormones in some way. In an almost throwaway phrase, she admonished, “We have all these expectations and things in our lives, and *somehow we don't learn how to manage this in a good way.”* Yet, managing the inflammatory conditions saturating our lives would be an incredible task; that it was suggested so casually reflects the dominant framing of health as one's personal responsibility and within one's control. Maya undertook an intercontinental move to do so, something not available to most people.

Many in endometriosis communities practice and advocate for “self‐management strategies” such as anti‐inflammatory diets. Diana, from the opening anecdote, avoided phytoestrogens in soy and xenoestrogens in detergents and plastic food packaging as she tinkered with symptom management. Self‐help books, courses, and podcasts often discuss managing endometriosis and other menstrual‐related conditions like premenstrual syndrome/tension (PMS/T), polycystic ovarian syndrome (PCOS), or premenstrual dysphoric disorder (PMDD) through “hormonal wellness.” One of my interviewees, Barbara, made the drastic decision to mitigate stress by cutting ties with her family, whom she found unsupportive to the extent that her pain was unbearable. “Management” of these sorts points towards the aspirational or partially effective application of the exposure model and how difficult it is to conceive of action outside individual agency. Yet, consider how Barbara, after reducing exposure to her family, poured her energy into cultivating a supportive endometriosis community. Although she did not discuss this as having anything to do with “the environment,” it might well be considered an ecological practice. Such a conceptual shift away from individuals and protection, from exposures and towards mutuality and cultivation of the good, is needed to operate within an embodied ecologies model.

## SLIPPERY KNOWLEDGE

It is ironic that in the opening vignette, Diana articulated what I am claiming was pushed to the margins or omitted. Likewise, the quotes from Mary and Maya show where environment *did* come up in my interviews. Rather than attempt to illustrate an omission, which is trickier (although perhaps more ethnographically admirable!), I share these people's musings as instances not of ignorance proper, but of what I am calling “slippery” knowledge: a conviction that evades action. Knowledge about toxins and environmental influences on health is slippery because it intersects and overlaps with other patterns of ignorance, notably those that reinforce gender, race, and class hierarchies. Nancy Tuana and Shannon Sullivan describe recognizing these patterns as “feminist epistemologies of ignorance” (2006); their work examines ignorance about female orgasms and women's health, alongside ignorance that bolsters race, racism, and White privilege (Sullivan & Tuana, 2007). What is considered known and knowable is inextricable from power.

Gender and race power dynamics create durable channels into which knowledge (and ignorance) is funneled. Such patterns of ignorance are related to the dearth of research, funding, and awareness about endometriosis itself, in that women's suffering is relatively accepted. Likewise, endometriosis is less acknowledged among racialized and poor people—the idea of endometriosis as a “career woman's disease,” which Čapek (2000) thought was being supplanted by environment/body connections, was always referencing a White, middle‐ or upper‐class woman, long the only social group for whom working could be considered optional. Eugenic encouragement for wealthy White women to reproduce was key to endometriosis being construed as a disease from which non‐White people did not suffer (Bougie et al., 2019), and Black women continue to receive poorer care for the condition (Perro et al., 2023). Dismissal of pain, whether as imagined or unimportant, taps into colonial narratives of White women as “overcivilized” and hysterical and Black women as “savage” and tough, narratives that shaped gynecology at its origins (Briggs, 2000). It is not a coincidence that women, minoritized genders, racialized, and poor people fare worse from environmental toxicity (e.g., Marya & Patel, 2021).

Instead of endometriosis being approached as a condition of systemic inflammation and an embodied reaction to diffuse environmental insults, power dynamics ingrained in habits of thought cause the disease to be approached through a womb‐centric lens. The ancient Greek idea of hysteria, a catch‐all female condition in which a “wandering womb” needed to be weighed down with child, is still present in how both pregnancy and hysterectomy are persistently called “cures” for endometriosis, as well as how women are still seen as unreliable narrators of their own experience and subordinate to the whims of their reproductive capacities (Kapsalis, 2017; Koerber, 2018). Wombs are still associated with disgust, taboo, and incomprehensibility, perpetuating ignorance from menstrual stigma to the properties of placentas (Guidone, 2020; Heyes, 2016; Owen, 2022). Some biomedical researchers are pushing against this in the way they brand their labs, emphasizing the remarkable regenerative and immune properties of the uterus, endometrium, and placenta to advocate for their relevance to medical science's “big questions” (Gross, 2021). And yet, this undermines the importance of women's experience *as such*, compounding the problem of people with endometriosis finally receiving attention and sympathy when they want to conceive, but not when their daily experience is intolerable. The one area where environmental etiologies have found traction is also highly womb‐focused: research and policy on the developmental origins of health and disease (DOHaD) frames the (potentially) pregnant woman as a fetal environment (see Lappé et al., 2019). This has been critiqued by social scientists for subsuming the pregnant person's personhood to that of the (potential) child and loading her with the responsibility for what she cannot control (e.g., Sharp et al., 2018; Valdez, 2022).

Habits of knowing slip into well‐worn grooves of ignorance, of impotence, of focus; knowledge that calls for (inconceivable) alternative patterns of action slips away. Environmental injustices are “slippery” in this sense. Some communities and neighborhoods are designated sacrifice zones, and some bodies are readily dismissed as “damaged,” to the detriment of both their health and reproductive potential. Max Liboiron (2021) equates pollution with colonialism, and Arlene Geronimus (2023) shows that chronic stress from existing in a race‐stratified society prematurely ages the body and contributes to a swath of chronic diseases. Decolonial and anti‐colonial frames about mutuality, collective relational consciousness, imbrication with the material world, and ecological responsibility—they slip away (Tallbear, 2019). Metaphors of slippage feel too gentle to describe the violence of colonialism; my hope is that they describe the insidious seepage of this violence into knowledge practices that construe themselves as beneficent and apolitical, including amongst those often considered the beneficiaries of colonialism, such as a middle‐class White patient in the United Kingdom.

Ignorance of environmental or ecological etiologies is not a dearth of information, per se; it's a dearth of possibilities for action. Such knowledge seems elusive, impractical, and overwhelming. Framing endometriosis as a systemic inflammatory condition (as recent medical research attempts to do) is slippery because framing bodies as ecologically embedded systems is slippery. In early work describing MCS, Kroll‐Smith and Floyd (1997) point out how poorly it fits within the biomedical model of disease: MCS pushes against the idea of disease as personal and internal, positing that at any moment, someone's “relative state of illness or wellness is a function, in part, of the activities and practices of others” (1997, xiii). It defies the logic of individual behavior, responsibility, and cure. Although MCS, like endometriosis, will not claim lives, “it will lay claim to an alternative strategy for the construction of rational knowledge in late modern society” (ibid., xiii). Such an alternate construction of rational knowledge, one that does not begin and end with individuals, is what is missing from endometriosis research and treatment.

## CONCLUSION: IMPLICATIONS FOR ENDOMETRIOSIS FUTURES

The visibility of endometriosis is increasing. It is a poster child for the recent women's health plans in the Scottish, United Kingdom, and Australian parliaments, with other countries, including the United States and France, following suit. The international Social sciencE Endometriosis Network (SEEN) was founded in 2021 to provide a forum and network for the rapidly growing membership. Attention to the enormous women's health funding gap is featured in major journals like *Nature* (Smith, 2023). The EA defines endometriosis on its homepage as “a life‐altering hormone and immune system disease,” without reference to gender. The *Guardian* reported that endometriosis research is on “the cusp of a breakthrough” (Jackson, 2023), while the specialist call for a breakthrough mentioned above references “environmental insults” and “the increasingly evident mismatch between evolutionary legacy and modern society” (EIG, 2024, 372). The Vanderbilt endometriosis studies found that a *father's* toxicant exposure can affect his daughter at conception, pushing against maternal‐centric approaches to transgenerational wellness (VUMC, 2023). It is an interesting time, building momentum that might—perhaps—shift the register of possible action.

What would it look like to reimagine endometriosis as an interlaced set of relations that exceeds dominant ways of apprehending bodies and worlds? One start might be to de‐center the goal of objectivity, which decades of feminist science studies have shown is not only unachievable but obscures a deeper worldview that shapes what is understood as possible to know and to do. The “Reframing Endometriosis” conference brought people who don't often communicate—eminent clinicians, social scientists, patient advocates, and activists from LGBTQ+, Black, and disability communities—into the same room to sit on sofas and listen to one another. A recurring theme was the need to decolonize endometriosis, though grasping the relevance of political concerns to disease was “slippery” for those more ingrained in a scientific mindset. More radical than acknowledging multiple “situated knowledges” (Haraway, 1988) is developing and acting from “desirable biases”—not aiming to be politically neutral but to advance a vision of justice in and via science. What would a bias towards anti‐colonial and anti‐patriarchal frames look like? An ecological view of disease is central to this question.
